# Co-contamination by heavy metal and organic pollutant alters impacts of genotypic richness on soil nutrients

**DOI:** 10.3389/fpls.2023.1124585

**Published:** 2023-01-26

**Authors:** Lin Huang, Si-Mei Yao, Yu Jin, Wei Xue, Fei-Hai Yu

**Affiliations:** ^1^ Zhejiang Provincial Key Laboratory of Evolutionary Ecology and Conservation/Institute of Wetland Ecology & Clone Ecology, Taizhou University, Taizhou, China; ^2^ College of Ecology and Environment, Chengdu University of Technology, Chengdu, Sichuan, China; ^3^ School of Ecology and Nature Conservation, Beijing Forestry University, Beijing, China

**Keywords:** cadmium, cypermethrin, complementarity, functional traits, genotypic diversity, pesticides, productivity, *Hydrocotyle vulgaris*

## Abstract

Co-contamination by heavy metal and organic pollutant may negatively influence plant performance, and increasing the number of genotypes for a plant population may reduce this negative effect. To test this hypothesis, we constructed experimental populations of *Hydrocotyle vulgaris* consisting of single, four or eight genotypes in soils contaminated by cadmium, cypermethrin or both. Biomass, leaf area and stem internode length of *H. vulgaris* were significantly lower in the soil contaminated by cypermethrin and by both cadmium and cypermethrin than in the soil contaminated by cadmium only. A reverse pattern was found for specific internode length and specific leaf area. In general, genotypic richness or its interaction with soil contamination did not influence plant growth or morphology. However, soil nutrients varied in response to soil contamination and genotypic richness. Moreover, plant population growth was positively correlated to soil total nitrogen, but negatively correlated to total potassium and organic matter. We conclude that co-contamination by cadmium and cypermethrin may suppress the growth of *H. vulgaris* population compared to contamination by cadmium only, but genotypic richness may play little role in regulating these effects.

## Introduction

The soil is one of the world’s most important resources, playing a crucial role in supporting the lives of humans, animals, plants and other living organisms (Bardgett and van der Putten, 2014; Bahram et al., 2018; Geisen et al., 2019; Guerra et al., 2020). However, due to rapid industry development and intense agriculture, soils in many ecosystems have been polluted, severely threating the health and sustainability of ecosystems (Nouri et al., 2009; He et al., 2014; Bandowe et al., 2019; Wang et al., 2019; Titaley et al., 2020; Alengebawy et al., 2021; Ali et al., 2022). As one of the most common pollutants in the soil, heavy metals have attracted global attentions (Chibuike and Obiora, 2014; Cristaldi et al., 2017; Shahid et al., 2017; Kumar et al., 2019; Eijsackers et al., 2020; Alengebawy et al., 2021). For example, cadmium can be taken up by plants and its accumulation in plant organs can inhabit plant growth due to toxicity (Raskin et al., 1994; Podar et al., 2004; Shahid et al., 2017; Wang et al., 2019). Soils are also frequently influenced by organic pollutants introduced by, e.g., oil exploitation and utilization of pesticides (Reid et al., 2000; Cristaldi et al., 2017; Sharma et al., 2019; Tudi et al., 2021). In particular, pesticides may persist in the soil for a long time and thus may be harmful to plants as they can alter the ecological process such as photosynthesis, enzyme activity, root growth and leaf formation (Boutin et al., 2014; Parween et al., 2016; Yadav and Devi, 2017; Aveek et al., 2019; Siddiqui et al., 2022). More often, soils in nature are co-contaminated by heavy metals and organic pollutants, which may enhance the negative effect of single pollutant on plant growth due to additive or synergic effects (Singh et al., 2017; Khudur et al., 2018; Zhao et al., 2018; Alengebawy et al., 2021; Ali et al., 2022). Therefore, it is crucial to test the response of plants to soil co-contaminated by heavy metals and organic pollutants to guide phytoremediation.

Biodiversity experiments have generally revealed a positive relationship between plant species richness and community productivity (e.g., Loreau and Hector, 2001; Tilman et al., 2001; van Ruijven and Berendse, 2003). These positive relationships are attributed to complementarity effect, i.e., more diverse communities can utilize resources more complementarily *via* niche differentiation and species facilitation, and/or selection effect, i.e., more diverse communities have a higher chance to include productive species (Loreau and Hector, 2001; Polley et al., 2003; van Ruijven and Berendse, 2005; Godoy et al., 2020). Therefore, increasing the number of species may potentially help the recovery of plant communities in contaminated soils. Similarly, increasing genotypic richness of a plant population may also promote the complementary utilization of resources and increase probability of the presence of particularly productive genotypes, as different genotypes of the same species also vary in their abilities to acquire resources (Fridley et al., 2007; Lankau and Strauss, 2007; Hughes et al., 2008; Baron et al., 2015). Thus, increasing genotypic richness of a plant population may increase its productivity (Crutsinger et al., 2006; Lankau and Strauss, 2007; Fridley and Grime, 2010; Kotowska et al., 2010; Schöb et al., 2015; Begum et al., 2022) and consequently the ability to remediate the contaminated soil. However, how genotypic richness influences the population productivity in soil co-contaminated by heavy metals and organic pollutants was largely unknown.

*Hydrocotyle vulgaris* is an introduced species in the Araliaceae family, and occurs in many natural and semi-natural wetlands and grasslands in China (Dong et al., 2015). Different genotypes of *H. vulgaris* can co-exist in the field, but often only one genotype is dominant (Wang et al., 2020). Previous studies have shown that *H. vulgaris* exhibits rapid asexual reproduction ability, high degree of phenotypic plasticity and strong tolerance ability (Dong, 1995; Dong et al., 2015; Liu et al., 2021; Xue et al., 2022). It has been used in the water purification for the removal of heavy metals and organic pollutants (Morand et al., 2011; Vafaei et al., 2013; Ni et al., 2018; Li et al., 2020). Therefore, to explore the population growth of *H. vulgaris* regulated by genotypic richness in contaminated soils may have significant implications for the application of this species in remediation of these contaminated soils. However, whether *H. vulgaris* can thrive in the soil co-contaminated by heavy metals and organic pollutants and thus may potentially be used for the remediation of contaminated soils is largely unknown.

To answer these questions, we conducted a greenhouse experiment by planting *H. vulgaris* populations consisted of one, four or eight genotypes in soil contaminated by cadmium, cypermethrin or both. Specifically, we tested the following hypothesis: (1) plant populations grown in the soil co-contaminated by cadmium and cypermethrin produces lower biomass than that grown in the soil contaminated by either cadmium or cypermethrin, due to additive or synergic effects of the two pollutants (Singh et al., 2017); (2) plant populations with more genotypes produces more biomass than those with less genotypes, due to higher utilization efficiency of soil nutrients; (3) the negative effect of co-contamination is weaker in populations of greater genotypic richness compared to those with lower genotypic richness.

## Materials and methods

### The species

*Hydrocotyle vulgaris* L. (Araliaceae) is a perennial amphibious plant. More than 30 years ago, this species was introduced into China as an ornamental species. This species can produce creeping stems, and newly produced ramets consisting of a leaf and some adventitious roots can emerge from the stem nodes (Dong, 1995; Xue et al., 2022). *H. vulgaris* is now widely distributed in many habitats as it can expand its distribution ranges *via* high phenotypic plasticity and rapidly vegetative growth (Dong et al., 2015; Huang et al., 2022).

In 2016, we collected ramets of *H. vulgaris* from 10 sites in different provinces in China (**Table 1**; Wang et al., 2020). Then we extracted total genomic DNA for the mature leaves of each collected ramet and detected their DNA methylation status using methylation-sensitive amplified polymorphism (MSAP) markers (see Wang et al., 2020 for more details). The ramet of different genotypes varies a lot in their phenotypic characteristics (Wang et al., 2020). Ramets of different genotypes were cultivated in separate containers and the newly produced ramets were collected from these containers and used in the experiment described below.

**Table 1:** Sampling cites of the initial ramets of *H. vulgaris* for genotype identification.

Sampling site	Longitude	Latitude	No. of ramets sampled/No. of genotypes identified in each population	ID of genotypes used in the experiment
Shenzhen	114°03′03″E	22°33′25″N	10/1	
Wenzhou	120°45′38″E	27°58′29″N	15/5	WZ-6, WZ-7
Lishui	119°49′09″E	28°23′11″N	10/2	LS-3
Taizhou	121°25′37″E	28°40′22″N	15/4	TZ-8
Chongqing	106°37′41″E	29°33′34″N	9/4	CQ-2
Ningbo	121°33′32″E	29°53′50″N	15/4	NB-3
Hangzhou	120°23′25″E	30°18′48″N	13/4	HZ-13
Wuhan	114°25′10″E	30°32′55″N	10/3	WH-1,WH-9
Jiaxing	120°46′28″E	30°41′55″N	21/1	JX-22
Shanghai	121°26′30″E	31°08′58″N	10/1	

### The experiment

The experiment consisted of three soil contamination treatments (i.e., soil was contaminated by cadmium, cypermethrin or both), crossed with three genotypic richness treatments (i.e., monoculture, 4-genotype and 8-genotype mixtures). We collected a field soil from a hill in Taizhou, Zhejiang Province, China. The soil was air-dried, sieved (2 cm mesh) and mixed evenly with sand at a volume ratio of 1:1 (the “bulk” soil hereafter). To generate the cadmium treatment, we added 5 ml solution of CdCl_2_·2.5H_2_O into 7 kg bulk soil, and filled them into a pot (27 cm in diameter and 18 cm in height; concentration of Cd equals 10 mg/kg). The concentration of Cd applied in the experiment was larger than the maximum concentration (~ 6.6 mg/kg) detected in the local contaminated soil. To generate the cypermethrin treatment, we added 14 ml synthetic pyrethroid pesticides (4.5% cypermethrin; Shandong Henglida Biotechnology Co., Ltd.) into 7 kg bulk soil, and filled them into a pot. The pesticide concentration applied in the experiment was 20 times as that applied in Tejada et al. (2015). In the co-contamination treatment, we added 5 ml solution of CdCl_2_·2.5H_2_O and 14 ml synthetic pyrethroid pesticides into 7 kg bulk soil, and filled them into a pot. Each soil contamination treatment had 29 pots; therefore, there were 3 contaminated soils × 29 pots = 78 pots in total.

Two weeks later, we planted *H. vulgaris* populations with different genotypic richness (i.e., monoculture, 4-genotype and 8-genotype mixtures) in these soils based on a field investigation (Wang et al., 2020). We used 10 genotypes of *H. vulgaris* to create the plant populations in this experiment. In monocultures, we planted 16 ramets of the same genotype in a pot, resulting in a total of 30 populations (pots) (10 genotypes × 3 contaminated soils). In the 4-genotype mixtures, we randomly selected four genotypes from the pool of the ten genotypes, and eight different combinations were randomly selected from a total of 210 ( $C_{10}^{4}$ ) combinations. A total of 24 populations (pots) (8 combinations × 3 contaminated soils) were created, and for a given 4-genotype combination we planted four ramets of each genotype in a pot. Similarly, in the 8-genotype mixtures, we first randomly selected eight genotypes from the genotype pool, and a total of eight different combinations were selected. A total of 24 8-genotype populations were created and each population consisted of two ramets of each genotype. In this study, we manipulated the number of genotypes but not specific composition of the population. Therefore, there were ten replicates for monocultures, eight replicates for the 4- and 8-genotype mixtures; and each replicate in the genotypic richness levels had a different, randomly determined combination of genotypes (**Table 2**; Tilman et al., 2001).

**Table 2:** Genotype combination used for different genotypic richness levels.

	Genotypic richness																									
Genotype ID	1										4								8							
NB-3																										
WZ-6																										
LS-3																										
JX-22																										
HZ-13																										
WH-1																										
CQ-2																										
WH-9																										
WZ-7																										
TZ-8																										
Replicate^1^	1	1	1	1	1	1	1	1	1	1	1	1	1	1	1	1	1	1	1	1	1	1	1	1	1	1

**^1^** Each population had one replicate for each genotype combination. Therefore, there were 10 (1 × 10 genotypes) replicates for monocultures, 8 (1 × 8 combinations) replicates for the 4-genotype and 8-genotype mixtures.

The size of the initial ramets used in the experiment were standardized and each ramet had a node with some adventitious roots, a petiole of 2 cm long, a proximal and a distal internode of 1 cm long. Ramets that were not established during the first two weeks of the experiment were replaced. All pots were watered regularly. The experiment was maintained for 90 days (22 July to 22 October 2020). During the experiment, the daily mean temperature was 27.1°C.

### Harvest and measurements

At the end of the experiment, we first counted the number of ramets in each pot. Then, for each pot, we harvested all ramets of different genotypes together, and separated them into roots, creeping stems and leaves. Biomass was obtained after being oven-dried at 80 °C for at least 48 h. During harvest, the root was washed over a 0.5 mm-mesh sieve. We also randomly selected 10 fully developed leaf blades and 10 mature internodes in each pot, and measured leaf area, internode length and their dry weight. Based on these data, we calculated specific leaf area (SLA) and specific internode length (SIL).

We also collected a subsample (200 g) of soil from each pot. The soil sample was air-dried and sieved (100 meshes) for the determination of soil total nitrogen (TN), soil total phosphorus (TP), soil total potassium (TK) and soil organic matter. We first added 5 ml of H_2_SO_4_ and 0.5 ml of HClO_4_ to the soil sample (0.5 g), digesting it at 360°C for 35 min, and then filtered the solution at room temperature (20°C). Soil TN and TP were determined by the spectrophotometric method with a continuous flow automated analyzer (Seal, Germany), and soil TK was determined by inductively coupled plasma-optical emission spectrometry (ICP-OES, Optima 2100 DV, Perkin Elmer, USA). Soil organic matters were measured by potassium dichromate volumetric method.

### Data analysis

We used linear mixed-effect models fitted with the *nlme* package (version 3.1-145; Pinheiro et al., 2018) to examine the effect of soil contamination treatment and genotypic richness. In the model, soil contamination treatment (cadmium vs. cypermethrin vs. co-contamination), genotypic richness (monoculture vs. 4-genotype vs. 8-genotype) and the interaction was used as fixed effects. The genotypic composition was included as a random effect. The response variables were the growth (total biomass, leaf biomass, creeping stem biomass, root biomass and number of ramets) and morphological traits (internode length, leaf area, SIL and SLA) of the population, and soil chemistry (TN, TP, TK and organic matter). *Post hoc Tukey’s HSD* tests were conducted to compare means.

We further tested the relationship between plant performance (growth and morphology) and soil chemistry using a stepwise multiple linear regression with backward selection procedure.

All analyses were performed with R (version 3.4.4; http://www.r-project.org) in RStudio (version 1.1.423; http://rstudio.org). Residuals of all variables were graphically checked for normality and homogeneity of variance.

## Results

### Population growth and morphology

**Figure 1:**
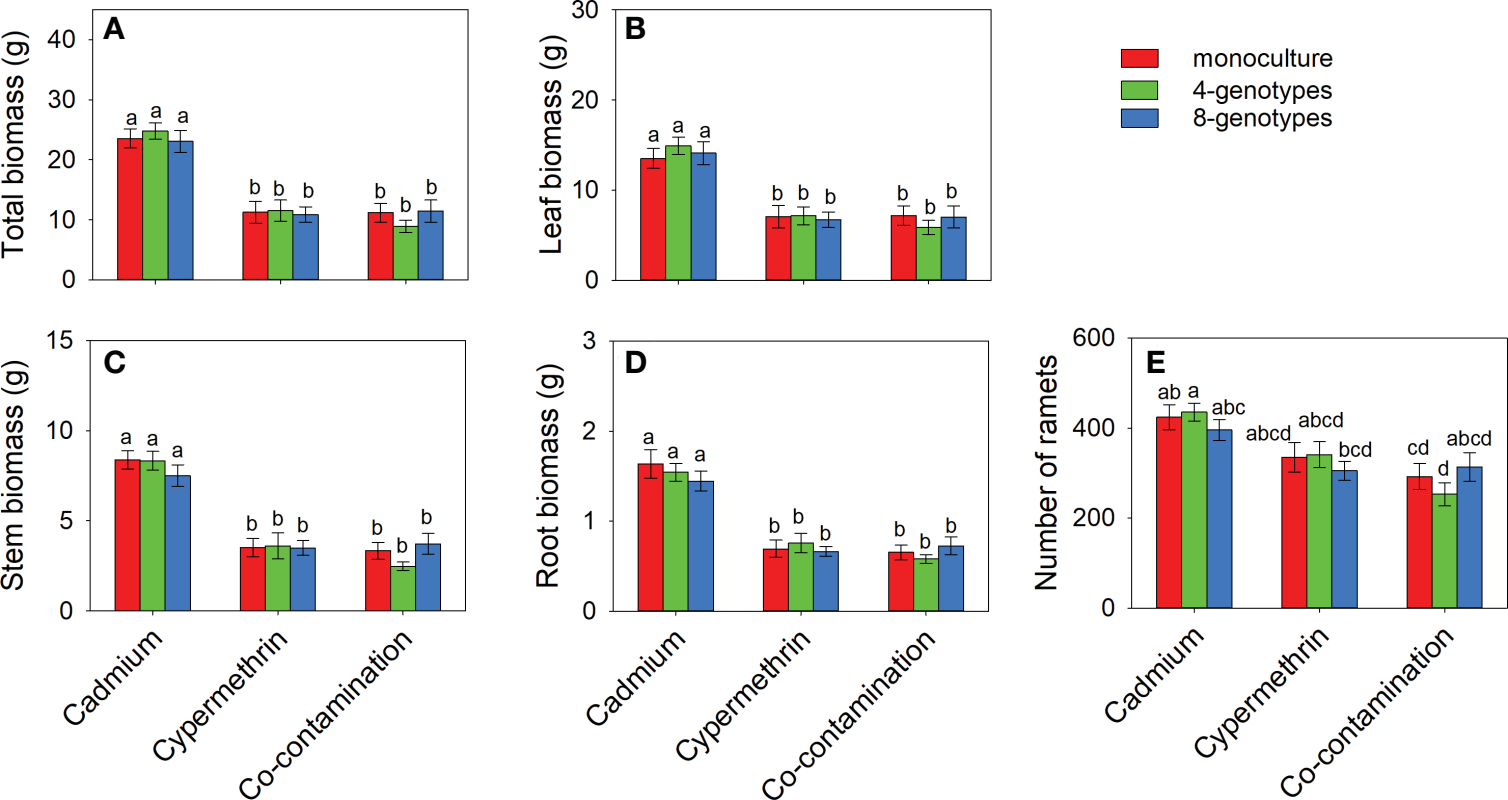
Total biomass **(A)**, leaf biomass **(B)**, stem biomass **(C)**, root biomass **(D)** and number of ramets **(E)** of *Hydrocotyle vulgaris* population consisting of different number of genotypes under different pollutant treatments. Mean values ( ± 1 SE) are presented. Letters (a-d) above the bars indicate significant differences (*P <* 0.05) among each panel. See **Table 1A** for ANOVA results.

**Figure 2:**
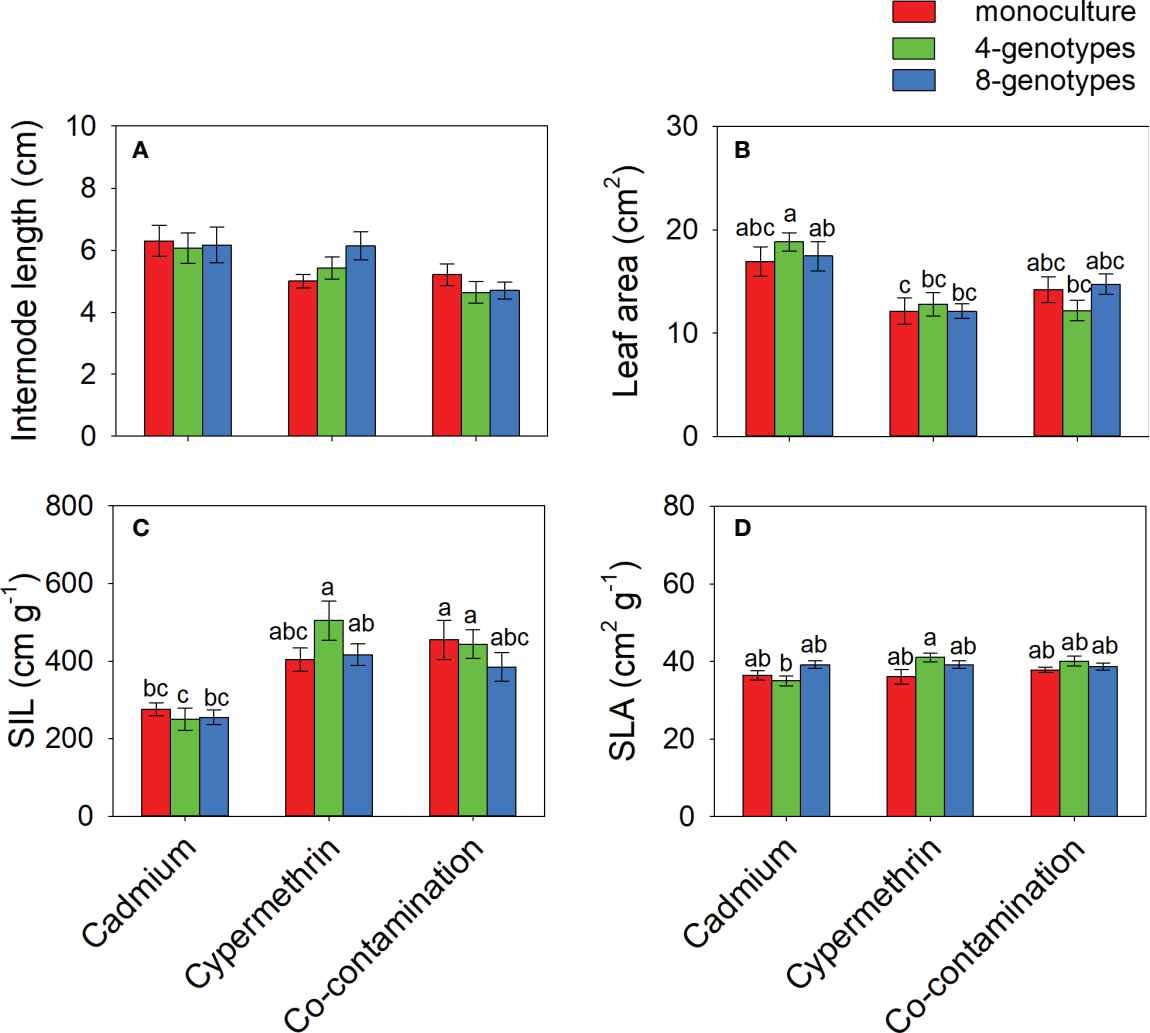
Internode length **(A)**, leaf area **(B)**, specific internode length (**C**; SIL) and specific leaf area (**D**; SLA) of *Hydrocotyle vulgaris* population consisting of different number of genotypes under different pollutant treatments. Mean values ( ± 1 SE) are presented. Letters (a-c) above the bars indicate significant differences (*P <* 0.05) among each panel. See **Table 1B** for ANOVA results.

Total biomass, leaf biomass, stem biomass, root biomass and number of ramets of *H. vulgaris* population in the soil contaminated by cadmium were overall greater (total biomass: 23.80 ± 1.60; leaf biomass: 14.18 ± 1.11; stem biomass: 8.08 ± 0.54; root biomass: 1.54 ± 0.12; number of ramets: 418.53 ± 23.43) than that in the soil contaminated by cypermethrin (total biomass: 11.23 ± 1.62; leaf biomass: 6.98 ± 1.03; stem biomass: 3.54 ± 0.55; root biomass: 0.70 ± 0.09; number of ramets: 326.98 ± 27.57) or co-contaminated by cadmium and cypermethrin (total biomass: 10.53 ± 1.47; leaf biomass: 6.70 ± 1.02; stem biomass: 3.18 ± 0.43; root biomass: 0.65 ± 0.08; number of ramets: 286.28 ± 28.94; **Figure 1** and **Table 3A**). However, genotypic richness or its interaction with contamination treatment did not influence the growth of *H. vulgaris* population (**Figure 1** and **Table 3A**). A similar pattern was also observed for internode length and leaf area (**Figures 2A, B** and **Table 3B**). SIL of the population was overall lower in the soil contaminated by cadmium (260.10 ± 21.80) than in the soil contaminated by cypermethrin (441.48 ± 36.17) or co-contaminated by cadmium and cypermethrin (427.98 ± 41.82; **Figure 2C** and **Table 3B**), and a similar tendency was also observed for SLA (**Figure 2D** and **Table 3B**).

**Table 3:** Results of linear-mixed models for effects of pollutant treatment, number of genotypes and the interaction on (A) growth and (B) morphology of *Hydrocotyle vulgaris* and (C) soil chemistry.

	Contamination treatment		Genotypic richness		Interaction	
	*F*_2, 47_	*P*	*F*_2, 22_	*P*	*F*_4, 47_	*P*
(A) Plant growth						
Total biomass	**64.24**	**<0.001**	0.02	0.980	0.50	0.733
Leaf biomass	**44.76**	**<0.001**	<0.01	0.997	0.44	0.777
Stem biomass	**83.61**	**<0.001**	0.22	0.801	1.03	0.404
Root biomass	**82.85**	**<0.001**	0.13	0.877	0.79	0.540
Number of ramets	**18.51**	**<0.001**	0.17	0.849	1.00	0.416
(B) Plant morphology						
Internode length	**7.84**	**0.001**	0.36	0.703	1.19	0.329
Leaf area	**19.22**	**<0.001**	0.04	0.961	1.10	0.366
SIL	**24.63**	**<0.001**	1.17	0.330	1.22	0.316
SLA	2.46	0.096	2.45	0.110	**2.70**	**0.042**
(C) Soil chemistry						
TN	**9.27**	**<0.001**	0.43	0.657	**6.24**	**<0.001**
TP^1^	**21.67**	**<0.001**	**6.10**	**0.008**	1.74	0.157
TK	**12.08**	**<0.001**	**12.92**	**<0.001**	**33.89**	**<0.001**
Organic matter	**4.79**	**0.013**	3.41	0.051	1.99	0.112

Numbers are values of F and P which are in bold when P < 0.05; ^1^ Data were log-transformed.

### Soil chemistry

**Figure 3:**
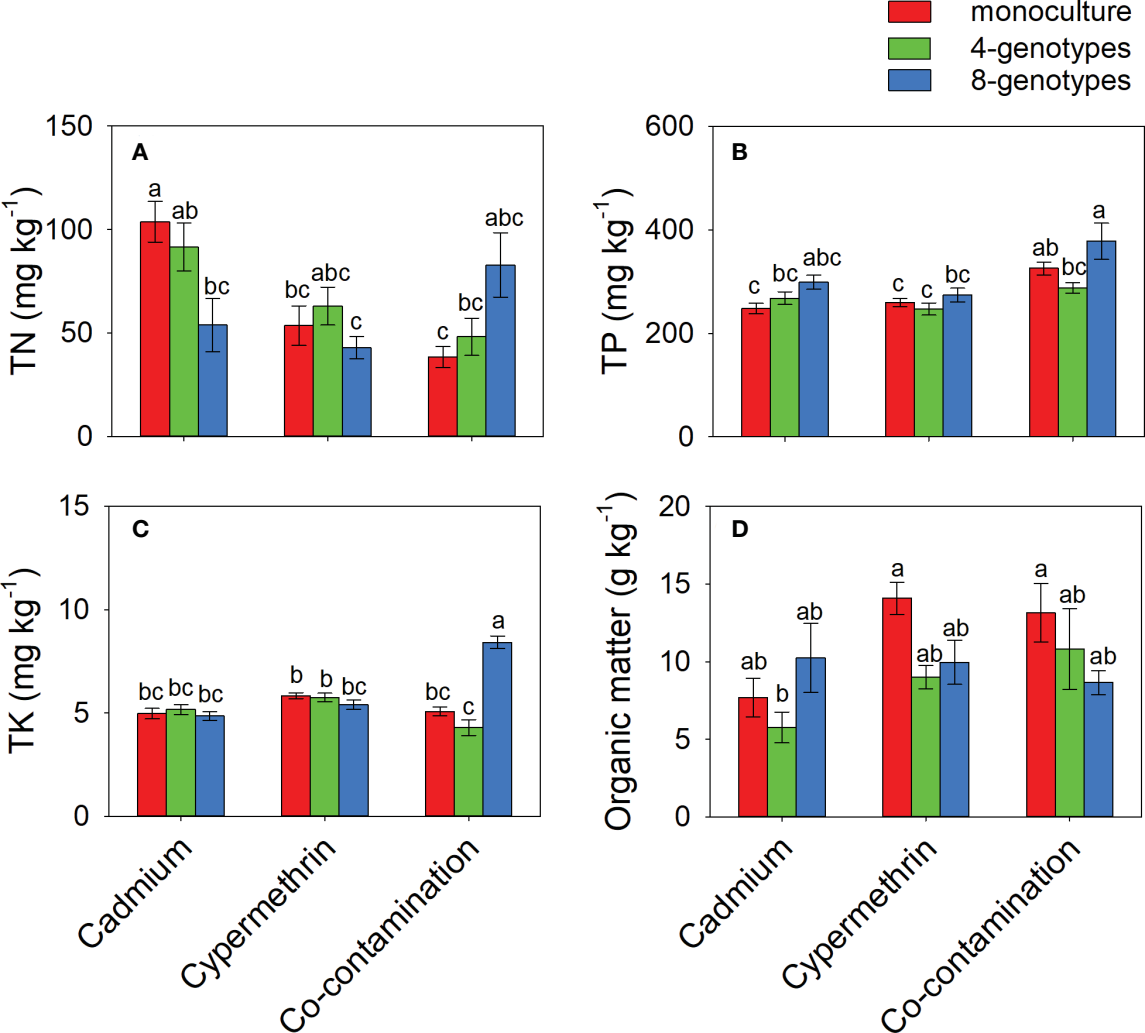
Total nitrogen (**A**; TN), total phosphorus **(B)**, total potassium (**C**; TP) and organic matters **(D)** in the soil grown with *Hydrocotyle vulgaris* populations of different number of genotypes under different pollutant treatments. Mean values ( ± 1 SE) are presented. Letters (a-c) above the bars indicate significant differences (P < 0.05) among each panel. See **Table 1C** for ANOVA results.

TN in the soil contaminated by cadmium was greater when the soil had grown with monocultures (103.77 ± 9.81) than when the soil had grown with 8-genotype mixtures (53.91 ± 12.87), but no difference was found in the soil contaminated by cypermethrin or co-contaminated by cadmium and cypermethrin (**Figure 3A** and **Table 3C**). TP was overall greater in the soil co-contaminated by cadmium and cypermethrin (330.43 ± 19.17) than in the soil contaminated by either cadmium (271.48 ± 12.02) or cypermethrin (260.05 ± 11.01), and also greater in the soil that had grown with 8-genotype mixtures (316.99 ± 20.69) than in the soil that had grown with monocultures (277.51 ± 10.10) and 4-genotype mixtures (267.46 ± 11.41; **Figure 3B** and **Table 3C**). TK in the soil co-contaminated by cadmium and cypermethrin was greater when the soil had grown with 8-genotype mixtures (8.42 ± 0.30) than when the soil had grown with monocultures (5.07 ± 0.22) or 4-genotype mixtures (4.30 ± 0.38), but no difference was found in the soil contaminated by either cadmium or cypermethrin (**Figure 3C** and **Table 3C**). Organic matter was generally lower in the soil contaminated by cadmium (7.89 ± 1.47) than in the soil contaminated by cypermethrin (11.01 ± 1.07) or co-contaminated by cadmium and cypermethrin (10.86 ± 1.76; **Figure 3D** and **Table 3C**).

### Relationship between population performance and soil chemistry

In general, plant growth was positively correlated to TN (all *P* < 0.001) but negatively correlated to TK (*P* = 0.001 – 0.006) and organic matter (*P* = 0.059 - 0.027) in the soil (**Table 4A**). Moreover, leaf area was positively correlated to soil TN (*P* < 0.001; **Table 4B**). Specific internode length was positively correlated to organic matter (*P* = 0.013) but negatively correlated to total nitrogen (*P* < 0.001; **Table 4B**).

**Table 4:** Relationship of the (A) growth and (B) morphology of *Hydrocotyle vulgaris* with soil chemical characteristics.

	(A) Plant growth					(B) Plant morphology			
	Total biomass	Leaf biomass	Stem biomass	Root biomass	Number of ramets	Internode length	Leaf area	SIL	SLA
TN	↑32.05^***^	↑27.08^***^	↑34.02^***^	↑28.59^***^	↑29.16^***^	3.27	↑15.91^***^	↓12.71^***^	–
TP	–	–	–	–	–	–	–	–	2.13
TK	↓8.88^**^	↓8.05^**^	↓7.88^**^	↓10.81^**^	–	2.00	–	–	–
Organic matter	↓4.29^*^	3.680	↓4.20^*^	↓5.07^*^	–	–	2.820	↑6.44^*^	–

F- and P-values were obtained from stepwise multiple liner regressions with backward selection procedure. Significance of relationships: ^***^P < 0.001, ^**^P < 0.01 and ^*^P < 0.05. “-” indicates variables that were not included in the final model. “↑” and “↓” indicate positive and negative relationships, respectively.

## Discussions

In this study, we show that co-contamination by cadmium and cypermethrin reduced growth of *H. vulgaris* population, compared to contamination by cadmium only. However, increasing the number of genotypes of the population did not alter this effect. Despite that, co-contamination by cadmium and cypermethrin altered soil nutrients depending on the number of genotypes. These results indicate that genotypic richness may play little role in helping *H. vulgaris* in the soil co-contaminated by cadmium and cypermethrin.

We observed a lower plant growth in the soil co-contaminated by cadmium and cypermethrin than in the soil contaminated by cadmium, indicating that co-contamination may suppress the growth of *H. vulgaris* (**Figure 1**). This result supported our first hypothesis partly, as we did not observe a growth difference in the co-contaminated soil and the soil contaminated by cypermethrin only. Therefore, the presence of cypermethrin may be the main driver of the negative effect of co-contamination in our study. As a pesticide mainly used for controlling insects, cypermethrin may also have directly negative influences on plant growth (Chauhan et al., 1999; Parween et al., 2016; Aveek et al., 2019). In this study, we observed that *H. vulgaris* population had a smaller leaf area (i.e., lower light capture ability) and a shorter, thinner internode (i.e., a poor escaping ability from harsh environment) in the two soils with cypermethrin. Besides, cypermethrin may also influence plant growth indirectly *via* changing soil abiotic and biotic characteristics (Gundi et al., 2007; Zhuang et al., 2011; Jia et al., 2019; Zhu et al., 2022). In our study, soil nitrogen was generally lower in the two soils with cypermethrin (**Figure 3A**), which may be associated with the poor plant growth in the two soils. Soil biota is also sensitive to cadmium and cypermethrin (Zhuang et al., 2011; Tejada et al., 2015; Liu et al., 2020; Zhu et al., 2022). The accumualtion of cadmium and cypermethrin in the soil can reduce the activity and diveristy of soil microbes, consequently reduce the performance of plants (Al-Ani et al., 2019; Jia et al., 2019; Cheng et al., 2022). However, we were unable to disentangle these effects in our study, as we did not measure soil biotic properties in this study.

We hypothesized a positive effect of genotypic richness on the growth of *H. vulgaris* population, as increasing the number of genotypes may promote the complementary utilization of resources and increase the occurrence probability of productive genotypes (Crutsinger et al., 2006; Hughes et al., 2008; Kotowska et al., 2010; Begum et al., 2022). However, we found that genotypic richness did not influence plant growth in this study and a similar result has also been reported in several other studies (Fridley and Grime, 2010; Prieto et al., 2015; Schöb et al., 2015). Our soil analysis revealed that increasing genotypic richness reduced total nitrogen in the soil contaminated by cadmium (**Figure 3A**), indicating that plant populations with greater genotypic richness have greater efficiency of nitrogen utilization than that with lower genotypic richness. However, the effect of genotypic richness on soil nitrogen did not alter the growth of *H. vulgaris*, despite that plant growth was significantly positively correlated to soil nitrogen (**Table 4**). Therefore, genotypic richness may help the removal of nitrogen but cannot promote the growth of *H. vulgaris* in the contaminated soil.

As the effect of co-contamination may have overwhelmed the effect of genotypic richness, it is not surprising that increasing the number of genotypes failed to reduce the negative effect of co-contamination on *H. vulgaris* populations. Hence, our third hypothesis was not supported. Soil contamination may have acted as a “filter” that drives the convergence of *H. vulgaris* populations (Laliberté et al., 2014; Kraft et al., 2015). Our analysis confirmed it by showing that plant populations with different genotypic richness had consistent morphology in the contaminated soils (**Figure 2**).

## Conclusions

In conclusion, co-contamination by cadmium and cypermethrin can suppress the growth of *H. vulgaris* population compared to contamination only by cadmium, but increased genotypic richness cannot help to reduce this negative effect. Although we detected a neutral genotypic richness effect, it does not mean that increasing genotypic diversity is not beneficial for the remediation of contaminated soils. It should be noted that the relative abundance of genotypes may be more important than the number of genotypes in driving the positive diversity effects at local scales, which we failed to investigate in this study (Huang et al., 2022). Therefore, we recommend a more diverse plant population/community by considering both the number of genotypes/species, and their relative abundance in the remediation of contaminate soils, as highly diverse populations/communities are typically more stable than less diverse ones in facing the rapid global changes such as soil contaminations (Tilman et al., 2006; Isbell et al., 2015). We also acknowledged that the result of our short-term experiment is not comparable to long-term ones since both soil pollutants and plant population structures may change over time due to complex plant-soil interactions. Hence, further studies on long-term plant responses to soil contamination may be crucial for guiding the remediation of contaminated soils.

## Data availability statement

The raw data supporting the conclusions of this article will be made available by the authors, without undue reservation.

## Author contributions

WX and F-HY designed the study. LH, S-MY, and YJ conducted the experiment and collected the data. LH and WX analyzed the data. LH, WX, and F-HY wrote the first version of the manuscript. All authors commented on and reviewed the final draft. All authors contributed to the article and approved the submitted version.
